# Sensitive Detection of 8-Nitroguanine in DNA by Chemical Derivatization Coupled with Online Solid-Phase Extraction LC-MS/MS

**DOI:** 10.3390/molecules23030605

**Published:** 2018-03-08

**Authors:** Chiung-Wen Hu, Yuan-Jhe Chang, Jian-Lian Chen, Yu-Wen Hsu, Mu-Rong Chao

**Affiliations:** 1 Department of Public Health, Chung Shan Medical University, Taichung 402, Taiwan; windyhu@csmu.edu.tw; 2 Department of Occupational Safety and Health, Chung Shan Medical University, Taichung 402, Taiwan; handsom1005@gmail.com (Y.-J.C.); yayen0619@gmail.com (Y.-W.H.); 3 School of Pharmacy, China Medical University, Taichung 404, Taiwan; cjl@mail.cmu.edu.tw; 4 Department of Optometry, Da-Yeh University, Changhua 515, Taiwan; 5 Department of Occupational Medicine, Chung Shan Medical University Hospital, Taichung 402, Taiwan

**Keywords:** online solid-phase extraction, LC-MS/MS, peroxynitrite, nitrated DNA lesion, derivatization, isotope-dilution

## Abstract

8-Nitroguanine (8-nitroG) is a major mutagenic nucleobase lesion generated by peroxynitrite during inflammation and has been used as a potential biomarker to evaluate inflammation-related carcinogenesis. Here, we present an online solid-phase extraction (SPE) LC-MS/MS method with 6-methoxy-2-naphthyl glyoxal hydrate (MTNG) derivatization for a sensitive and precise measurement of 8-nitroG in DNA. Derivatization optimization revealed that an excess of MTNG is required to achieve complete derivatization in DNA hydrolysates (MTNG: 8-nitroG molar ratio of 3740:1). The use of online SPE effectively avoided ion-source contamination from derivatization reagent by washing away all unreacted MTNG before column chromatography and the ionization process in mass spectrometry. With the use of isotope-labeled internal standard, the detection limit was as low as 0.015 nM. Inter- and intraday imprecision was <5.0%. This method was compared to a previous direct LC-MS/MS method without derivatization. The comparison showed an excellent fit and consistency, suggesting that the present method has satisfactory effectiveness and reliability for 8-nitroG analysis. This method was further applied to determine the 8-nitroG in human urine. 8-NitroG was not detectable using LC-MS/MS with derivatization, whereas a significant false-positive signal was detected without derivatization. It highlights the use of MTNG derivatization in 8-nitroG analysis for increasing the method specificity.

## 1. Introduction

Chronic inflammation has been linked to heart disease, obesity, diabetes and cancer [1,2]. Under chronic inflammatory conditions, exuberant NO production by activated macrophages is believed to be an important tissue-damage mediator as NO can be further converted into several highly reactive species, such as nitrous anhydride, nitrogen dioxide, nitryl chloride and peroxynitrite [3].

Peroxynitrite is a relatively stable reactive species with a half-life of ~one second at physiological pH and can penetrate the nucleus and induce damage in DNA [4,5]. 8-Nitroguanine (8-nitroG) is the first identified peroxynitrite-mediated nitration product. The formation of 8-nitroG is generally rationalized in terms of addition of low reactive •NO_2_ to the highly oxidizing guanine radical that results from the deprotonation of guanine radical cation initially generated by one-electron oxidation of guanine [6]. 8-NitroG formed in DNA is chemically unstable and can spontaneously depurinate, yielding apurinic sites with the resultant possibility of GC-to-TA mutation [7]. Alternatively, adenine can be preferentially incorporated opposite 8-nitroG during DNA syntheses, resulting in GC-to-TA transversion [8]. Several research groups have focused on the role of 8-nitroG in infection- and inflammation-related carcinogenesis and examined the formation of this lesion in laboratory animals and clinical samples [9,10]. Their studies have shown that the 8-nitroG formation occurred to a much greater extent in cancerous tissue than in the adjacent non-cancerous tissue and that its formation increased with inflammatory grade [11,12,13], suggesting that 8-nitroG could be a potential biomarker of inflammation-related carcinogenesis [12]. 

In the past decade, cellular 8-nitroG levels have been largely semi-quantitatively measured by immunohistochemistry [14,15,16] or quantitatively measured by HPLC with electrochemical detection (ECD) [17,18]. For quantitative measurement, the reported HPLC-ECD methods had comparatively high detection limits of 20–1000 fmol/injection [19,20] and required the reduction of 8-nitroG by a reducing agent (i.e., the reduction of 8-nitroG to 8-aminoguanine) by sodium hydrosulfite [21], which results in low reproducibility owing to the varied reaction efficiencies.

LC-MS/MS has received a great deal of attention in recent years because it can provide a sensitive and selective means for comprehensive measurement of multiple DNA lesions. In our previous work [22], we demonstrated that 8-nitroG is unstable and readily depurinates with a short half-life (e.g., 2.4 h in double-stranded DNA and 1.6 h in single-stranded DNA at 37 °C). We therefore proposed an LC-MS/MS method for the direct measurement of 8-nitroG in DNA and provided a strategy to overcome the chemical instability of 8-nitroG for the quantitative analysis of cellular 8-nitroG. However, this method was hampered by insufficient sensitivity, and the 8-nitroG was not retained well on the reversed-phase columns, decreasing the separation efficiency.

In this study, we describe a chemical derivatization coupled with online solid-phase extraction (SPE) LC-MS/MS analysis for the sensitive determination of 8-nitroG in DNA. To investigate its effectiveness and reliability, the present method was further compared to direct LC-MS/MS measurement without chemical derivatization [22]. 

## 2. Results

### 2.1. LC-MS/MS Characteristics of 8-NitroG-MTNG

**Figure 1:**
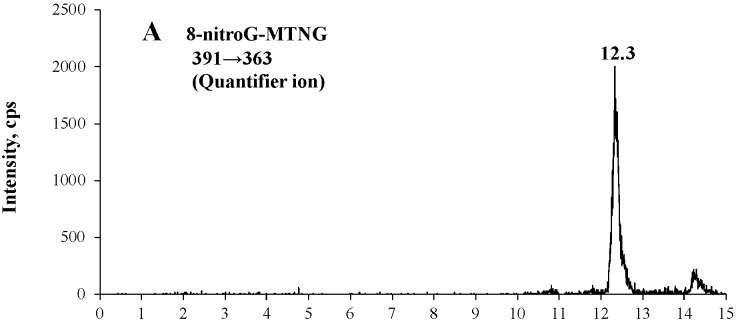

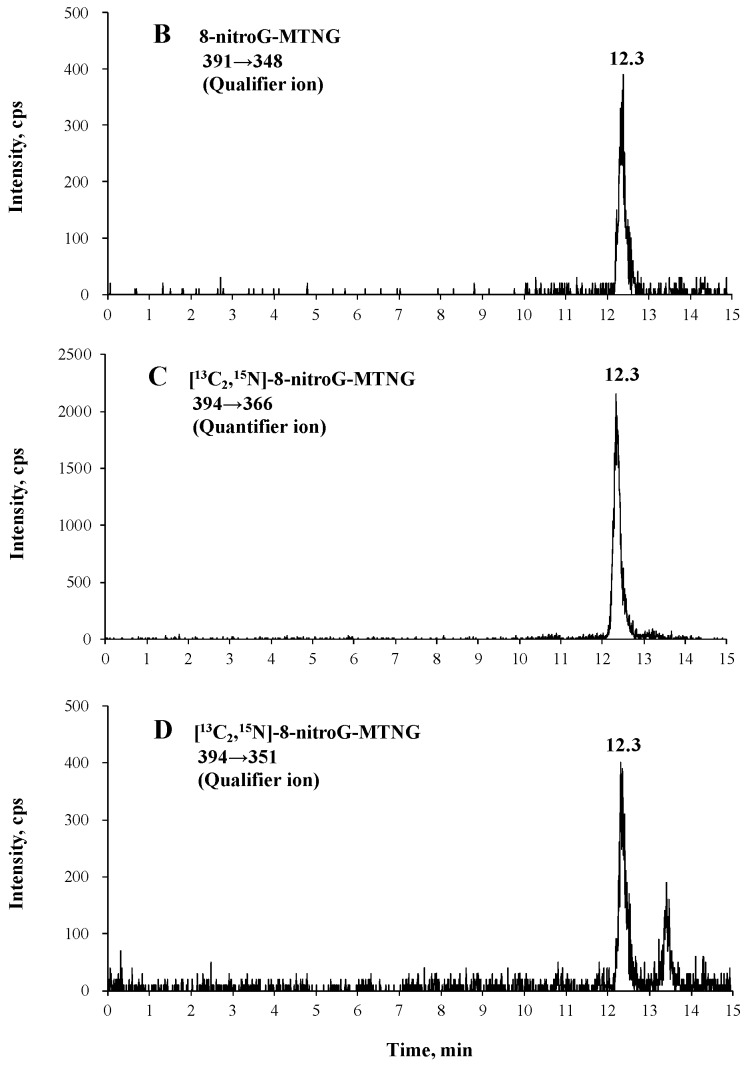
Chromatograms of 8-nitroG-MTNG in a hydrolysate of calf thymus DNA that had been treated with 10 μM peroxynitrite, as measured by LC-MS/MS coupled with online SPE. 8-NitroG-MTNG was monitored at *m*/*z* 391→363 (**A**) and *m*/*z* 391→348 (**B**), and the internal standard [^13^C_2_,^15^N]-8-nitroG-MTNG was monitored at *m*/*z* 394→366 (**C**) and *m*/*z* 394→351 (**D**). cps, counts per second.

Figure 1 shows an example chromatogram of 8-nitroG-MTNG and its isotope internal standard [^13^C_2_,^15^N]-8-nitroG-MTNG of a calf thymus DNA hydrolysate that had been treated with 10 μM ONOO^−^. The retention times of 8-nitroG-MTNG and [^13^C_2_,^15^N]-8-nitroG-MTNG are concordant. Low background noise from the biological matrix showed the good selectivity of the method. The negative ESI mass spectrum of 8-nitroG-MTNG contained a [M − H]^−^ precursor ion at *m*/*z* 391 and product ions at *m*/*z* 363 (quantifier ion, Figure 1A) and *m*/*z* 348 (qualifier ion, Figure 1B) due to loss of CO or C_2_H_3_O; a precursor ion at *m*/*z* 394 and product ions at *m*/*z* 366 (quantifier ion, Figure 1C) and *m*/*z* 351 (qualifier ion, Figure 1D) characterized the [^13^C_2_,^15^N]-8-nitroG-MTNG.

### 2.2. Optimization of Derivatization Reaction with MTNG

**Figure 2:**
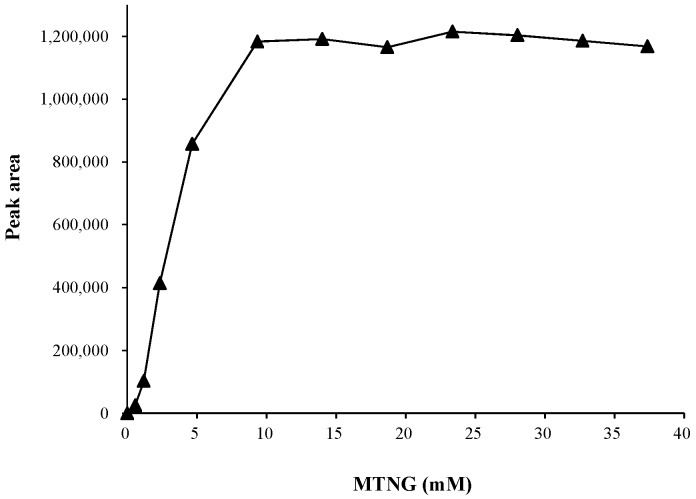
Effects of the concentration of added MTNG on the derivatization yield. The peak areas of 8-nitroG-MTNG obtained from the derivatization of a hydrolysate of calf thymus DNA containing 1 μM 8-nitroG. Points denote the mean values of duplicates.

We investigated the yields for the formation of the conjugate at different molar ratios of MTNG to 8-nitroG (from 232:1 to 14,960:1). As shown in Figure 2, the amounts of 8-nitroG formed increased in a dose-dependent manner with increasing MTNG concentration (0.58–9.35 mM). The derivatization was efficient when the ratio reached 3740:1, where MTNG was added at 9.35 mM. However, we used an even higher MTNG concentration (14.0 mM) for derivatization in this study to ensure a complete derivatization reaction in light of the high variability of biological matrices. This suggested that 5.6 μmole MTNG/μg DNA is needed.

### 2.3. Method Validation

The limit of quantification (LOQ) was defined as the lowest 8-nitroG-MTNG sample concentration meeting prespecified requirements for precision and accuracy within 20%. Using the present method, the LOQ was determined in DNA hydrolysates to be 0.05 nM. The limit of detection (LOD), defined as the lowest concentration that gave a signal-to-noise ratio of at least 3 in DNA hydrolysates, was found to be 0.015 nM (0.3 fmol, see Supplementary Materials, Figure S1), which corresponds to 0.15 μmol 8-nitroG/mol of guanine when using 50 μg of DNA per analysis.

The calibration curve consisted of seven calibration points from 0.16 to 10.3 pmol, and each calibrator contained 0.25 pmol [^13^C_2_,^15^N]-8-nitroG. The resulting peak area ratios (analyte to internal standard) were plotted against the corresponding pmol (Supplementary Material, Figure S2). Linear regression calculations were unweighted and non-zero-forced, and the regression equation was calculated as y = 3.9531x + 0.061. The observed correlation coefficients (R^2^) during validation were consistently greater than 0.999. All of the calibrators fell within 5% deviation of back-calculated concentrations from nominal spiked concentrations, with an imprecision (CV) < 10%. Meanwhile, the peak identity of 8-nitroG-MTNG in DNA hydrolysates was confirmed by comparing the peak area ratios (quantifier ion/qualifier ion) with those of the calibrators. As an acceptance criterion, ratios in DNA samples should not deviate by more than ±25% from the mean ratios in the calibrators.

The intraday and interday imprecisions was determined from the analysis of three independent DNA samples, which were respectively treated with 50, 100 and 200 μM peroxynitrite. Intraday imprecision was estimated within one batch by analyzing six replicates, whereas interday imprecision was estimated on six separate occasions occurring over a period of 10 days. As shown in Table 1, intraday imprecision was determined to be 1.0–2.7%, and interday imprecision was 2.0–2.5%.

Recovery was evaluated by adding unlabeled standard mixture at five different levels to three peroxynitrite-treated DNA samples and measuring three replicates of these samples. As shown in (Supplementary Material, Figure S3), the mean recoveries were 96–104%, 99–105% and 96–104%, respectively, for those three DNA samples as estimated from the increase in the measured amount after addition of the analyte divided by the amount added, while the recoveries as calculated from the slope of the regression were 103%, 98% and 103% (R^2^ > 0.99), respectively.

Matrix effects were calculated according to the following equation:

(1) $$\mathrm{Matrix}\;\mathrm{effects}=1-\left(\frac{\mathrm{Peak}\;\mathrm{area}\;\mathrm{of}\;\mathrm{internal}\;\mathrm{standard}\;\mathrm{in}\;\mathrm{the}\;\mathrm{presence}\;\mathrm{of}\;\mathrm{matrix}}{\mathrm{Peak}\;\mathrm{area}\;\mathrm{of}\;\mathrm{internal}\;\mathrm{standard}\;\mathrm{in}\;\mathrm{the}\;\mathrm{absence}\;\mathrm{of}\;\mathrm{matrix}}\right)\times 100\%$$

The peak area in the presence of matrix refers to the peak area of the internal standard in DNA samples, while the peak area in the absence of matrix refers to the peak area of the internal standard prepared in deionized water. The relative change in the peak area of the internal standard was attributed to matrix effects, which reflect the combination of reduced derivatization efficiency, online extraction loss and ion suppression due to the DNA matrix. In this study, the matrix effects for 8-nitroG-MTNG were less than 20% in all DNA samples. Although the use of stable isotope-labeled internal standards could have compensated for different matrix effects, the low matrix effect achieved in this study ensures the high sensitivity of the method.

### 2.4. 8-NitroG in Calf Thymus DNA Treated with Peroxynitrite

The present method was applied to quantify the levels of 8-nitroG in calf thymus DNA, which was incubated with various concentrations (2.5–200 μM) of peroxynitrite. 8-NitroG was not detected in control DNA. In the peroxynitrite-treated samples, the levels of 8-nitroG increased in a dose-dependent manner with peroxynitrite concentration (Supplementary Material, Figure S4). 8-NitroG was formed at a level of 211 μmol/mol of guanine even with a peroxynitrite concentration as low as 2.5 μM. The formation of 8-nitroG reached a maximum level of 5823 μmol/mol of guanine when treated with 200 μM peroxynitrite.

### 2.5. Comparison between 8-NitroG Analysis Using Online SPE LC-MS/MS Method with and without MTNG Derivatization

**Figure 3:**
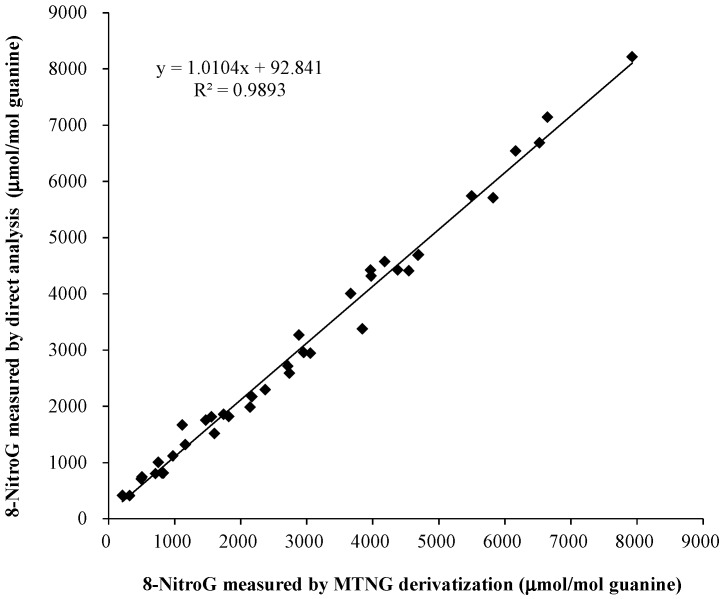
Correlation between quantitative results obtained through online SPE LC-MS/MS analyses with (this work) and without glyoxal derivatization [22]. The DNA samples containing various levels of 8-nitroG were prepared by treating calf thymus DNA with peroxynitrite at concentrations of 2.5–300 μM.

8-NitroG concentrations determined using the proposed online SPE LC-MS/MS following MTNG derivatization were compared to concentrations derived from the same samples using a direct online LC-MS/MS method without derivatization. As shown in Figure 3, regression analysis showed that the two methods were highly correlated (Pearson R² = 0.9893, *p* < 0.001, *n* = 39). The 8-nitroG levels derived from the present method were close to those obtained from the reported direct measurement by online SPE LC-MS/MS without derivatization, giving a slope of 1.01. 

## 3. Discussion

Despite numerous attempts, unambiguous evidence for the formation of 8-nitroG in cellular DNA or animal organs has not yet been provided. Apparently, the presence of 8-nitroG was detected by immunohistochemical methods in inflamed tissues [13]. However, the specificity of monoclonal/polyclonal antibodies against single oxidized base (such as 8-oxo-7,8-dihydroguanine) has been questioned due to the occurrence of cross-reactivity with overwhelming guanine bases [23]. The data provided by the several HPLC-ECD methods [19,20,21] that were aimed at measuring 8-nitroG, mostly as amino derivatives (8-aminoguaine), might not also be convincing. Therefore, there is a strong need of more accurate methods such as LC-MS/MS that appears to be the gold standard analytical tool for detecting DNA base lesions.

Our method coupling LC-MS/MS with derivatization and stable isotope-dilution facilitates the accurate and sensitive detection of cellular 8-nitroG. We further performed derivatization optimization, which is essential for an accurate measurement. The results revealed that an excess of MTNG is required (MTNG:8-nitroG molar ratio 3740:1, Figure 2) to completely derivatize 8-nitroG in DNA hydrolysates. This may be attributable to the fact that MTNG reacts not only with 8-nitroG but also with other guanine compounds present in biological samples [24]. 

Our method to estimate the cellular levels of 8-nitroG has some notable benefits compared with previously reported methods. The primary feature of our method is its high sensitivity (LOD: 0.015 nM), which may allow for the detection of extremely low levels of 8-nitroG in cellular DNA or urine. As shown in Supplementary Material, Figure S5, the sensitivity of the present method increased greatly with the derivatization, by approximately 10 times, compared to the method without derivatization (direct measurement [22]). Previously, Villaño et al. [25] and Wu et al. [26] developed HPLC-MS/MS methods for direct determination of 8-nitroG in plasma and urine, respectively, and reported LODs of 0.15–0.4 nM. One previous study by Ishii et al. [27] also attempted to measure 8-nitroG using LC-MS following MTNG derivatization. However, their method was not validated with a LOD (~1 nM) approximately 70 times higher than our method and a low specificity due to only the precursor ion was monitored. Meanwhile, it is also noted that that the MTNG amount used in the work of Ishii et al. [27] was significantly insufficient (0.09 μmole/μg DNA) for a complete derivatization reaction as compared to our finding (5.6 μmole MTNG/μg DNA). Furthermore, when comparing the LODs of previously reported chromatographic methods (except for LC-MS methods) for 8-nitroG, LODs of 2–100 nM were reported [17,20,28], and those are 100–6000 times higher than our LOD of 0.015 nM. Meanwhile, we have attempted to measure the background level of 8-nitroG in cellular DNA using the proposed method. The cells (human endothelial hybrid cells and Chinese hamster ovary cells) were lysed, subjected to acid hydrolysis [22], and derivatization with MTNG as described above. The 8-nitroG in cellular DNA was found to be non-detectable (see Supplementary Material Figure S6). It was estimated that background level of 8-nitroG in cellular DNA was less than 0.15 μmol/mol of guanine when using 50 μg of DNA per analysis. 

The second important feature of our method is the use of the isotope-dilution method for the 8-nitroG measurement, which permits high precision and accuracy. We added the stable isotope-labeled standard to the nucleoside mixtures; thus, the analytes and the corresponding internal standard were derivatized simultaneously with MTNG. Any variation/alteration in experimental conditions (e.g., derivatization efficiency, matrix effect and MS/MS performance variation) will thus be compensated for, and the accuracy of measurement will be ensured. Additionally, the co-elution of the analyte and its isotope-labeled standard along with the similar fragmentation pattern of the analyte and internal standard offers unequivocal chemical specificity for analyte identification [29]. A high accuracy measurement can also be supported by the observation of the high consistency (with a slope of 1.01, Figure 3) of the measured results with and without derivatization. The same strategy was also reported previously by Ishii et al. [27], who employed the stable isotope-labeled internal standard ([^13^C_2_,^15^N]-8-nitroG) in the LC-MS method for 8-nitroG measurement.

**Figure 4:**
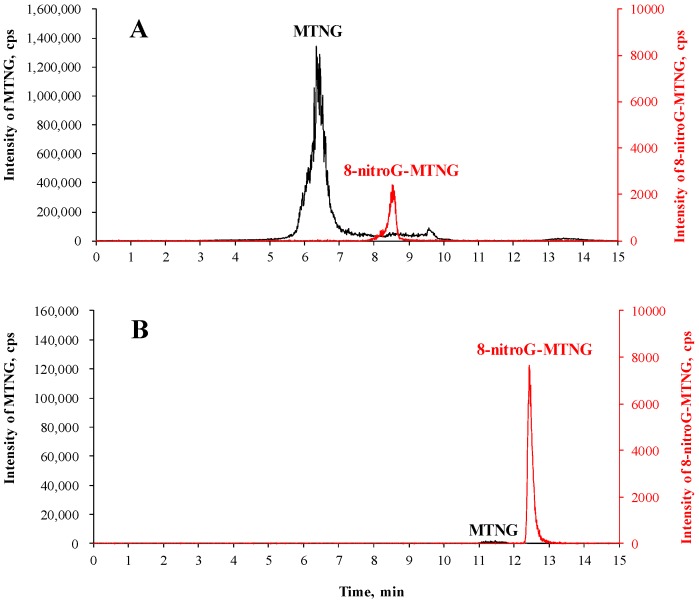
Total ion chromatogram of a derivatized DNA sample during trap column separation (**A**) and analytical column separation after column switching (**B**). MTNG was monitored at *m*/*z* 215→144 in positive ionization mode with a retention time at 6.3 min and 8-nitroG-MTNG were clearly well separated on the SPE column (**A**), and only the fraction containing 8-nitroG-MTNG at the retention time from 7.5 to 9.5 min was eluted into the analytical column (**B**).

The third advantage of the proposed method is the use of online SPE, which avoids ion-source contamination and significant matrix effects, resulting from the presence of a large amount of MTNG. To achieve complete derivatization, an excess of MTNG is required. However, part of this large excess could have remained unreacted and later injected into the LC-MS/MS system. As shown in Figure 4, the above problem was effectively avoided by the use of online SPE; since all unreacted MTNG was washed away during online SPE prior to analytical column chromatography and the ionization process in mass spectrometry. It was estimated that less than 0.01% of unreacted MTNG was introduced to the analytical column (Figure 4B).

Sawa et al. [30] measured 8-nitroG in urine using HPLC-ECD following immunoaffinity purification. They suggested that 8-nitroG in urine may be a potential biomarker of nitrative damage, although its level in urine could be as low as ~0.01 nM with a low detection rate. Interestingly, several recent studies from the same group have measured significantly high levels of 8-nitroG in the urine of healthy subjects (~4–3700 nM, [26,31,32]) as measured by LC-MS/MS. We presumed that these conflicting results in the literature could be attributed to the interference present in the urine samples as detected by a low-resolution mass spectrometer. To test our assumption, a serial measurement was conducted in 10 urine samples of healthy subjects; these samples were simultaneously measured by three different methods, including LC-MS/MS without derivatization, the present LC-MS/MS method with derivatization and UPLC-high-resolution MS (HRMS) (LTQ-Orbitrap Elite MS, Thermo Fisher Scientific). The UPLC gradient, column material and HRMS parameters applied is provided in Supplementary Material Table S1. A typical comparison of chromatograms of 8-nitroG in urine as measured by the three methods is given (Supplementary Material, Figure S7). A significant signal in a urine sample is noted to have the same transition *m*/*z* 195→178 as 8-nitroG and co-eluted with its internal standard (Supplementary Material, Figure S7A) as measured by LC-MS/MS without derivatization. However, when the same urine sample was measured by the present LC-MS/MS method with derivatization or UPLC-HRMS, no 8-nitroG was detected (Supplementary Material, Figure S7B,C). These findings proved that the urinary 8-nitroG signal detected by LC-MS/MS without derivatization (Supplementary Material, Figure S7A) was an interferent. In fact, the urinary 8-nitroG levels could be less than 0.01 nM in healthy subjects (<LOD of Supplementary Material, Figure S7B in urine). Meanwhile, it is worth noting that no 8-nitroG detected in urine by the present LC-MS/MS method with MTNG derivatization (Supplementary Material, Figure S7B) highlights the use of MTNG derivatization in 8-nitroG analysis for increasing the method specificity.

In conclusion, this study describes a sensitive and reliable LC-MS/MS method to quantitatively analyze 8-nitroG in DNA hydrolysates. With the combination of online SPE and MTNG derivatization, matrix interferences are significantly reduced, and the sensitivity of the present method has been highly increased. The present method was compared to a previous direct LC-MS/MS method without chemical derivatization. The comparison showed an excellent fit (R² = 0.9893, *p* < 0.001) and consistency (slope = 1.01), suggesting that the present method has satisfactory effectiveness and reliability for 8-nitroG analysis. More importantly, an excellent consistency proved that no artifact was produced during our derivatization that frequently encountered in the chemical derivatization of modified DNA bases [33]. Since 8-nitroG presents at trace levels in cells, our method could be useful in both laboratory and clinical research to understand the correlation between inflammation-related DNA damage and carcinogenesis. Subsequently, there is a limitation in the present work, which is the lack of other parameters optimization for MTNG derivatization, including the reaction buffers, reaction time, temperature, pH, etc.

## 4. Materials and Methods

### 4.1. Chemicals

8-NitroG (>98% purity) and [^13^C_2_,^15^N]-8-nitroguanine ([^13^C_2_,^15^N]-8-nitroG, >50% purity) were purchased from Toronto Research Chemicals (North York, Ontario, Canada). The purity and concentration of [^13^C_2_,^15^N]-8-nitroG was quantified by HPLC-UV using unlabeled 8-nitroG standards and confirmed by LC-MS/MS analysis. 6-Methoxy-2-naphthyl glyoxal hydrate (MTNG), diethylenetriaminepentaacetic acid (DTPA), peroxynitrite (ONOO^−^), calf thymus DNA, acetonitrile (ACN), dimethyl sulfoxide (DMSO) and ammonium acetate (AA) were obtained from Sigma-Aldrich (St. Louis, MO, USA). 

### 4.2. Stock and Working Solutions

Standard stock solutions of 8-nitroG and [^13^C_2_,^15^N]-8-nitroG were individually prepared in 5% (*v*/*v*) methanol at a concentration of 0.1 mM and stored at −20 °C. A series of standard working solutions of 8-nitroG (3.2–204 nM) were prepared by serial dilution of the stock solution with deionized water. The internal standard solution of [^13^C_2_,^15^N]-8-nitroG at a concentration of 5 nM was made by diluting the stock solution with deionized water. The MTNG stock solution was initially prepared by dissolving it in DMSO to a final concentration of 50 mM, after which it was protected from light and stored at −20 °C; it was diluted to the desired concentration with DMSO before use.

### 4.3. Nitration of Calf Thymus DNA by Peroxynitrite

The peroxynitrite (ONOO^−^) solution was carefully thawed and kept on ice. An aliquot of the stock solution was diluted 40-fold with 0.3 N NaOH, and the absorbance at 302 nm was measured with 0.3 N NaOH as blank. The peroxynitrite concentration was calculated using a molar absorption coefficient of 1670 M^−1^ cm^−1^. Fifty microliters of the peroxynitrite prepared in 0.3 N NaOH at various concentrations was added to 150 μL of reaction mixture that contained 50 μL of 6 μg/mL calf thymus DNA, 50 μL of 1 M AA (pH 7.4) and 50 μL of 1 mM DTPA (a metal chelator) in 0.3 N HCl. The sample was mixed by vortexing for 1 min at room temperature with a final pH at ~7.4. As the half-life of peroxynitrite is only 1–2 s near neutral pH, the reaction completed rapidly. Control experiments were performed using decomposed ONOO^−^ in NaOH, obtained by leaving the peroxynitrite solution overnight at room temperature, after which ONOO^−^ was completely decomposed as determined spectrophotometrically [34]. 

### 4.4. Hydrolysis and Derivatization of DNA Samples for 8-NitroG Analysis

**Figure 5:**
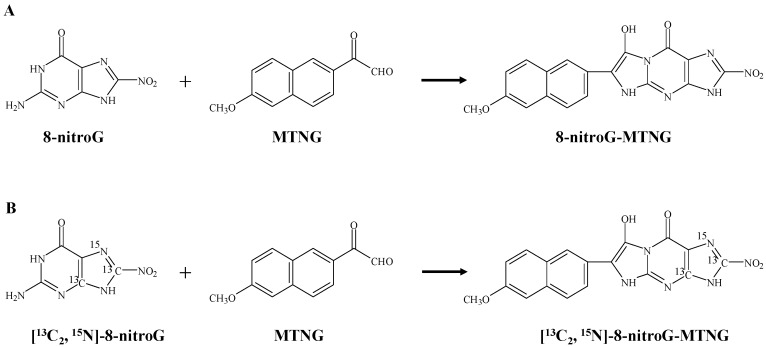
Derivatization of unlabeled 8-nitroG and [^13^C_2_,^15^N]-8-nitroG with MTNG to form 8-nitroG-MTNG (**A**) and [^13^C_2_,^15^N]-8-nitroG-MTNG (**B**).

Fifty-microliter DNA samples were spiked with 50 μL of 5 nM [^13^C_2_,^15^N]-8-nitroG and subjected to acid hydrolysis in 100 μL of 1 N HCl for 30 min at 80 °C, followed by neutralization with 100 μL of 1 N NaOH. 8-NitroG derivatization and reaction buffer used were described previously [24,27,35] and was performed with some modifications. Reaction buffer was prepared by combining 10 mL of 20 mM sodium acetate buffer (pH 4.8), 1 mL of 3 M sodium acetate buffer (pH 5.1) and 1 mL of 1 M Tris-HCl buffer (pH 8.0), aliquoted and stored at −20 °C. The samples were derivatized by mixing a portion of DNA hydrolysate (150 μL) with 60 μL of 14 mM MTNG, 150 μL of reaction buffer and 15 μL of 1 N HCl for 90 min at 25 °C to yield 8-nitroG-MTNG. The derivatized sample was transferred to a vial for online SPE LC-MS/MS determination. The chemical structures of 8-nitroG-MTNG and its corresponding internal standard ([^13^C_2_,^15^N]-8-nitroG-MTNG) are shown in Figure 5. To establish a linear calibration curve, 8-nitroG standards (3.2, 6.4, 12.7, 25.5, 51, 102 and 204 nM) in 50 μL of 6 μg/mL blank (untreated) DNA were mixed with 50 μL of 5 nM [^13^C_2_,^15^N]-8-nitroG and then hydrolyzed and derivatized as described above. The levels of 8-nitroG in DNA were expressed as μmol/mol of guanine. The analysis of guanine was performed by an isotope-dilution LC-MS/MS method previously described by Chao et al. [36].

### 4.5. Automated Online Extraction System and Liquid Chromatography

Column switching was controlled by a multi-channel valve (6-port, 2-position valve, VICI Valco, Houston, TX, USA) according to the pattern shown in detail in a previous publication [37]. An Agilent 1100 series HPLC system (Agilent Technologies, Wilmington, DE, USA) equipped with two binary pumps was used. The detailed column-switching operation sequence is summarized in Table 2. For online purification, an SPE column (33 × 2.1 mm i.d., 5 μm, Inertsil, ODS-3) was employed, while a reversed-phase C18 column (75 × 2.1 mm i.d., 5 μm, Inertsil, ODS-3) was used as the analytical column. The injection volume for the prepared DNA samples was 20 μL. After injection, the SPE column was loaded and washed for 7.5 min with Eluent I at a flow rate of 200 μL/min. After valve switching, 8-nitroG-MTNG were eluted to the analytical column with Eluent II at a flow rate of 200 μL/min. The valve was switched back to the starting position at 9.5 min; the SPE column was then reconditioned for the next run.

### 4.6. ESI-MS/MS

**Figure 6:**
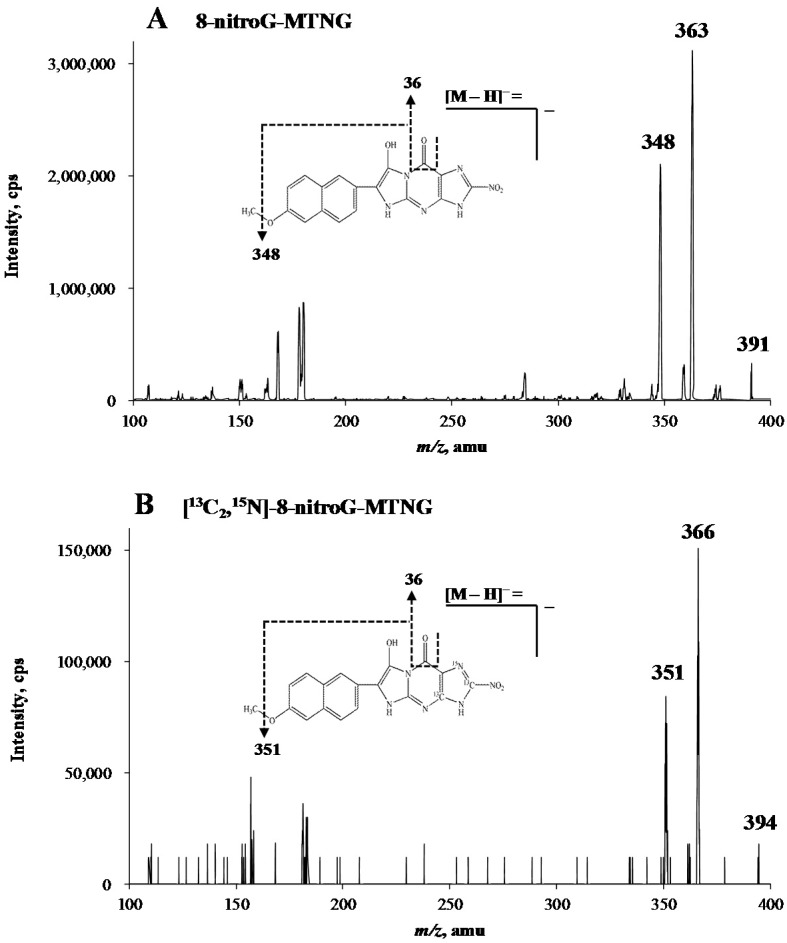
Negative ion electrospray MS/MS spectra of [M − H]^−^ of 8-nitroG-MTNG (**A**) and [^13^C_2_,^15^N]-8-nitroG-MTNG (**B**).

Mass spectrometric analysis was performed on an API 4000 QTrap hybrid triple quadrupole linear ion trap mass spectrometer (Applied Biosystems, Framingham, MA, USA) equipped with a TurboIonSpray (TIS) source. The resolution was set to a peak width (FWHM) of 0.7 Th for both Q1 and Q3 quadrupoles. Instrument parameters were optimized by infusion experiments with standard derivatives (8-nitroG-MTNG and [^13^C_2_,^15^N]-8-nitroG-MTNG) in negative ionization mode. Prior to infusion, the standard derivatives were purified by a manual C18 SPE to remove the salts; the standard derivatives were loaded onto a Sep-Pak C18 cartridge (100 mg/1 mL, Waters, Milford, MA, USA) preconditioned with methanol and deionized water. The cartridge was then washed with 1 mL of 20% methanol and eluted with 1 mL of 60% methanol. The eluate was suitable for precursor and product ion scan. Detailed product ion spectra of 8-nitroG-MTNG and its corresponding internal standard ([^13^C_2_,^15^N]-8-nitroG-MTNG) are given in Figure 6.

The ion spray voltage was maintained at −4500 V. The TIS source temperature was set at 450 °C. Ion source gas 1 (GS1) was set at 70 (arbitrary unit), ion source gas 2 (GS2) at 70, curtain gas at 10, and collision-activated dissociation gas at medium. Detection was performed in multiple reaction monitoring (MRM) mode. The precursor and product ions, along with optimized parameters, are given in Table 3. The most abundant fragment ion was used for quantification (quantifier ion), and the second most abundant ion was used for qualification (qualifier ion). Analyst 1.4.2 software (Applied Biosystems) was used for data acquisition and processing.

### 4.7. Optimization of MTNG Derivatization

The optimal amount of MTNG addition for derivatization was first investigated. The optimization test was performed by mixing 150 μL of DNA hydrolysate containing 1 μM 8-nitroG with 150 μL of reaction buffer, 15 μL 1 N HCl and 60 μL of various concentrations of MTNG (0.58–37.4 mM). The resulting mixture was then incubated at 25 °C for 90 min, followed by online SPE LC-MS/MS analysis.

### 4.8. Direct Measurement of 8-NitroG by Online SPE LC-MS/MS without Derivatization

The 8-nitroG levels in peroxynitrite-treated DNA were measured in parallel by a recently reported online SPE LC-MS/MS method without derivatization [22]. Briefly, the treated DNA samples (50 μL) were spiked with 50 μL of 10 ng/mL [^13^C_2_,^15^N]-8-nitroG, subjected to acid hydrolysis by adding 100 μL of 1 N HCl for 30 min at 80 °C, neutralized with 100 μL of 1 N NaOH and directly analyzed by online SPE LC-MS/MS. The samples were analyzed in the negative ion MRM mode. The 8-nitroG was monitored at *m*/*z* 195→178 (quantifier ion) and 195→153 (qualifier ion), and [^13^C_2_,^15^N]-8-nitroG was monitored at *m*/*z* 198→181.

## Figures and Tables

**Table 1:** Precision of isotope-dilution LC-MS/MS method with MTNG derivatization for analysis of 8-nitroG in DNA.

Characteristics for 8-nitroG ^a^	Sample 1	Sample 2	Sample 3
Intraday variation (pmol, mean ± SD) ^b^ (CV, %)	0.67 ± 0.02 (2.4)	0.90 ± 0.02 (2.7)	1.42 ± 0.01 (1.0)
Interday variation (pmol, mean ± SD) ^b^ (CV, %)	0.64 ± 0.02 (2.5)	0.90 ± 0.01 (2.0)	1.37 ± 0.03 (2.0)

^a^ 50 μL aliquots of 6 μg/mL calf thymus DNA were individually treated with peroxynitrite at three different concentrations (50 μM for sample 1, 100 μM for sample 2 and 200 μM for sample 3); ^b^ Each DNA solution was analyzed 6 times for the intraday and interday tests; the interday test was performed over a period of 10 days.

**Table 2:** Timetable for the column-switching procedure.

Time (min)	Eluent I (SPE Column)		Eluent II (Analytical Column)		Valve Position	Flow Rate (μL/min)	Remarks
	Solvent Ia ^a^ (%)	Solvent Ib ^b^ (%)	Solvent Iia ^a^ (%)	Solvent Iib ^b^ (%)			
0.0	70	30	50	50	A	200	Sample injection and washing
7.5	70	30	50	50	B	200	Start of elution of 8-nitroG-MTNG to the analytical column
9.5	70	30	50	50	A	200	End of elution; SPE column cleanup and reconditioning
10.0	70	30	50	50	A	200	
10.1	0	100	50	50	A	200	
10.5	0	100	0	100	A	200	
11.5	0	100	0	100	A	200	
12.0	70	30	50	50	A	200	
15.0	70	30	50	50	A	200	

^a^ 5% (*v*/*v*) ACN containing 1 mM AA; ^b^ 80% (*v*/*v*) ACN containing 1 mM AA.

**Table 3:** Tandem mass spectrometry parameters for 8-nitroG-MTNG and [^13^C_2_,^15^N]-8-nitroG-MTNG.

Compound	Q1 Mass (amu)	Q3 Mass (amu)	Dwell Time (ms)	DP ^a^ (V)	EP ^b^ (V)	CXP ^c^ (V)	CE ^d^ (V)
8-nitroG-MTNG	391	363 ^e^	100	−50	−11	−11	−30
	391	348	100	−50	−11	−11	−40
[^13^C_2_,^15^N]-8-nitroG-MTNG	394	366 ^e^	100	−50	−11	−11	−30
	394	351	100	−50	−11	−11	−45

^a^ Declustering potential; ^b^ Entrance potential; ^c^ Collision cell exit potential; ^d^ Collision energy; ^e^ Quantifier transition.

## Supplementary Materials

The Supplementary Materials are available online.

## Abbreviations

8-nitroG	8-Nitroguanine
LC-MS/MS	liquid chromatography-tandem mass spectrometry
LOD	limit of detection
LOQ	imit of quantification
MTNG	6-methoxy-2-naphthyl glyoxal hydrate
MRM	multiple reaction monitoring
ONOO^−^	peroxynitrite
SPE	solid-phase extraction
UPLC-HRMS	ultra-performance liquid chromatography-high resolution mass spectrometry

## References

[1] Fougere B., Boulanger E., Nourhashemi F., Guyonnet S., Cesari M. (2016). Chronic Inflammation: Accelerator of Biological Aging. J. Gerontol. A Biol. Sci. Med. Sci..

[2] El Assar M., Angulo J., Rodriguez-Manas L. (2013). Oxidative stress and vascular inflammation in aging. Free Radic. Biol. Med..

[3] Niles J.C., Wishnok J.S., Tannenbaum S.R. (2006). Peroxynitrite-induced oxidation and nitration products of guanine and 8-oxoguanine: Structures and mechanisms of product formation. Nitric Oxide.

[4] Beckman J.S., Chen J., Ischiropoulos H., Crow J.P. (1994). Oxidative chemistry of peroxynitrite. Methods Enzymol..

[5] Squadrito G.L., Pryor W.A. (1998). Oxidative chemistry of nitric oxide: The roles of superoxide, peroxynitrite, and carbon dioxide. Free Radic. Biol. Med..

[6] Cadet J., Wagner J.R., Shafirovich V., Geacintov N.E. (2014). One-electron oxidation reactions of purine and pyrimidine bases in cellular DNA. Int. J. Radiat. Biol..

[7] Ohshima H., Sawa T., Akaike T. (2006). 8-nitroguanine, a product of nitrative DNA damage caused by reactive nitrogen species: Formation, occurrence, and implications in inflammation and carcinogenesis. Antioxid. Redox Signal..

[8] Suzuki N., Yasui M., Geacintov N.E., Shafirovich V., Shibutani S. (2005). Miscoding events during DNA synthesis past the nitration-damaged base 8-nitroguanine. Biochemistry.

[9] Hiraku Y. (2014). Oxidative and nitrative DNA damage induced by environmental factors and cancer risk assessment. Fukuoka Igaku Zasshi.

[10] Sawa T., Ohshima H. (2006). Nitrative DNA damage in inflammation and its possible role in carcinogenesis. Nitric Oxide.

[11] Kawanishi S., Hiraku Y. (2006). Oxidative and nitrative DNA damage as biomarker for carcinogenesis with special reference to inflammation. Antioxid. Redox Signal..

[12] Kawanishi S., Ohnishi S., Ma N., Hiraku Y., Oikawa S., Murata M. (2016). Nitrative and oxidative DNA damage in infection-related carcinogenesis in relation to cancer stem cells. Genes Environ..

[13] Murata M., Thanan R., Ma N., Kawanishi S. (2012). Role of nitrative and oxidative DNA damage in inflammation-related carcinogenesis. J. Biomed. Biotechnol..

[14] Hiraku Y., Sakai K., Shibata E., Kamijima M., Hisanaga N., Ma N., Kawanishi S., Murata M. (2014). Formation of the nitrative DNA lesion 8-nitroguanine is associated with asbestos contents in human lung tissues: A pilot study. J. Occup. Health.

[15] Saigusa S., Araki T., Tanaka K., Hashimoto K., Okita Y., Fujikawa H., Okugawa Y., Toiyama Y., Inoue Y., Uchida K. (2013). Identification of patients with developing ulcerative colitis-associated neoplasia by nitrative DNA damage marker 8-nitroguanin expression in rectal mucosa. J. Clin. Gastroenterol..

[16] Kawanishi S., Hiraku Y., Pinlaor S., Ma N. (2006). Oxidative and nitrative DNA damage in animals and patients with inflammatory diseases in relation to inflammation-related carcinogenesis. Biol. Chem..

[17] Hsieh Y.S., Chen B.C., Shiow S.J., Wang H.C., Hsu J.D., Wang C.J. (2002). Formation of 8-nitroguanine in tobacco cigarette smokers and in tobacco smoke-exposed Wistar rats. Chem. Biol. Interact..

[18] Yermilov V., Rubio J., Ohshima H. (1995). Formation of 8-nitroguanine in DNA treated with peroxynitrite in vitro and its rapid removal from DNA by depurination. FEBS Lett..

[19] Chang H.R., Lai C.C., Lian J.D., Lin C.C., Wang C.J. (2005). Formation of 8-nitroguanine in blood of patients with inflammatory gouty arthritis. Clin. Chim. Acta.

[20] Ohshima H., Yoshie Y., Auriol S., Gilibert I. (1998). Antioxidant and pro-oxidant actions of flavonoids: Effects on DNA damage induced by nitric oxide, peroxynitrite and nitroxyl anion. Free Radic. Biol. Med..

[21] Tuo J., Liu L., Poulsen H.E., Weimann A., Svendsen O., Loft S. (2000). Importance of guanine nitration and hydroxylation in DNA in vitro and in vivo. Free Radic. Biol. Med..

[22] Hu C.W., Chang Y.J., Hsu Y.W., Chen J.L., Wang T.S., Chao M.R. (2016). Comprehensive analysis of the formation and stability of peroxynitrite-derived 8-nitroguanine by LC-MS/MS: Strategy for the quantitative analysis of cellular 8-nitroguanine. Free Radic. Biol. Med..

[23] Garratt L.W., Mistry V., Singh R., Sandhu J.K., Sheil B., Cooke M.S., Sly P.D. (2010). Arestcf, Interpretation of urinary 8-oxo-7,8-dihydro-2′-deoxyguanosine is adversely affected by methodological inaccuracies when using a commercial ELISA. Free Radic. Biol. Med..

[24] Katayama M., Matsuda Y., Kobayashi K., Kaneko S., Ishikawa H. (2006). Monitoring of 8-oxo-7,8-dihydro-2′-deoxyguanosine in urine by high-performance liquid chromatography after pre-column derivatization with glyoxal reagents. Biomed. Chromatogr..

[25] Villaño D., Vilaplana C., Medina S., Cejuela-Anta R., Martínez-Sanz J.M., Gil P., Genieser H.G., Ferreres F., Gil-Izquierdo A. (2015). Effect of elite physical exercise by triathletes on seven catabolites of DNA oxidation. Free Radic. Res..

[26] Wu C., Chen S.T., Peng K.H., Cheng T.J., Wu K.Y. (2016). Concurrent quantification of multiple biomarkers indicative of oxidative stress status using liquid chromatography-tandem mass spectrometry. Anal. Biochem..

[27] Ishii Y., Ogara A., Okamura T., Umemura T., Nishikawa A., Iwasaki Y., Ito R., Saito K., Hirose M., Nakazawa H. (2007). Development of quantitative analysis of 8-nitroguanine concomitant with 8-hydroxydeoxyguanosine formation by liquid chromatography with mass spectrometry and glyoxal derivatization. J. Pharm. Biomed. Anal..

[28] Li M.J., Zhang J.B., Li W.L., Chu Q.C., Ye J.N. (2011). Capillary electrophoretic determination of DNA damage markers: Content of 8-hydroxy-2′-deoxyguanosine and 8-nitroguanine in urine. J. Chromatogr. B Anal. Technol. Biomed. Life Sci..

[29] Pitt J.J. (2009). Principles and applications of liquid chromatography-mass spectrometry in clinical biochemistry. Clin. Biochem. Rev..

[30] Sawa T., Tatemichi M., Akaike T., Barbin A., Ohshima H. (2006). Analysis of urinary 8-nitroguanine, a marker of nitrative nucleic acid damage, by high-performance liquid chromatography-electrochemical detection coupled with immunoaffinity purification: Association with cigarette smoking. Free Radic. Biol. Med..

[31] Lin H.J., Chen S.T., Wu H.Y., Hsu H.C., Chen M.F., Lee Y.T., Wu K.Y., Chien K.L. (2015). Urinary biomarkers of oxidative and nitrosative stress and the risk for incident stroke: A nested case-control study from a community-based cohort. Int. J. Cardiol..

[32] Wang P.W., Chen M.L., Huang L.W., Yang W., Wu K.Y., Huang Y.F. (2015). Nonylphenol exposure is associated with oxidative and nitrative stress in pregnant women. Free Radic. Res..

[33] Dizdaroglu M. (1998). Facts about the artifacts in the measurement of oxidative DNA base damage by gas chromatography mass spectrometry. Free Radic. Res..

[34] Levrand S., Pesse B., Feihl F., Waeber B., Pacher P., Rolli J., Schaller M.D., Liaudet L. (2005). Peroxynitrite is a potent inhibitor of NF-κB activation triggered by inflammatory stimuli in cardiac and endothelial cell lines. J. Biol. Chem..

[35] Nakae D., Mizumoto Y., Kobayashi E., Noguchi O., Konishi Y. (1995). Improved genomic/nuclear DNA extraction for 8-hydroxydeoxyguanosine analysis of small amounts of rat liver tissue. Cancer Lett..

[36] Chao M.R., Wang C.J., Yen C.C., Yang H.H., Lu Y.C., Chang L.W., Hu C.W. (2007). Simultaneous determination of N7-alkylguanines in DNA by isotope-dilution LC-tandem MS coupled with automated solid-phase extraction and its application to a small fish model. Biochem. J..

[37] Hu C.W., Chao M.R., Sie C.H. (2010). Urinary analysis of 8-oxo-7,8-dihydroguanine and 8-oxo-7,8-dihydro-2′-deoxyguanosine by isotope-dilution LC-MS/MS with automated solid-phase extraction: Study of 8-oxo-7,8-dihydroguanine stability. Free Radic. Biol. Med..

