# Supplementary material for: Sensitive Detection of 8-Nitroguanine in DNA by Chemical Derivatization Coupled with Online Solid-Phase Extraction LC-MS/MS
Source: Molecules. 2018 Mar 8;23(3):605. doi: 10.3390/molecules23030605 (PMC6017919; doi:10.3390/molecules23030605)
Supplement: Supplementary file 1 [file molecules-23-00605-s001.pdf]

Supplementary Material

Sensitive detection of 8-nitroguanine in DNA by chemical derivatization coupled with online solid-phase extraction LC-MS/MS

Chiung-Wen Hu [1,†], Yuan-Jhe Chang [2,†], Jian-Lian Chen [3], Yu-Wen Hsu [2,4], and Mu-Rong Chao [2,5,*]

[1] Department of Public Health, Chung Shan Medical University, Taichung 402, Taiwan

[2] Department of Occupational Safety and Health, Chung Shan Medical University, Taichung 402, Taiwan

[3] School of Pharmacy, China Medical University, Taichung 404, Taiwan

[4] Department of Optometry, Da-Yeh University, Changhua 515, Taiwan

[5] Department of Occupational Medicine, Chung Shan Medical University Hospital, Taichung 402, Taiwan

* Corresponding author. Dr. Mu-Rong Chao, Department of Occupational Safety and Health, Chung Shan Medical University, No.110, Sec.1, Chien-Kuo N Road, Taichung 402, Taiwan. Tel.: +886-4-24730022, ext. 12116; fax: +886-4-23248194. E-mail address: chaomurong@gmail.com; mrchao@csmu.edu.tw.

† Chiung-Wen Hu and Yuan-Jhe Chang contributed equally to this work.

Table S1. LC-Orbitrap-MS chromatographic and mass spectrometric conditions used to determine 8-nitroG in urine.

UPLC parameters

Instrument: ACQUITY UPLC system, Waters

Colunm: Kinetex core-shell C18 column (100 mm x 2.1 mm I.D., 2.6 μm)

Injection volume: 5μL

Temperature: 45 ℃

Eluent gradient:

Time (min)	Mobile phase A[a] (%)	Mobile phase B[b] (%)	Flow rate (μL/min)
0.00	100	0	500
0.54	100	0	500
1.50	80	20	500
2.50	80	20	500
2.55	100	0	500
5.00	100	0	500

[a]Mobile phase A: deionized water containing 0.1% (v/v) formic acid.

[b]Mobile phase B: 50% (v/v) methanol containing 0.1% (v/v) formic acid

MS parameters

Instrument: LTQ-Orbitrap Elite MS, Thermo Fisher Scientific

Ionization parameters	Acquisition parameters
Mode: ESI negative ion	Resolution: 120,000
Source voltage: 3.2 kV	SRM (Selected reaction monitoring) scan
Capillary temperature: 360 ℃	Transition 1: 195.0266 > 177.95-178.05
Source heater temp: 350 ℃	Transition 2: 198.0304 > 180.95-181.05
Sheath gas: 30	Collision mode: HCD (100 eV)
Aux gas: 15	Isolation width (*m/z*): 2
S-Lens RF: 60%	Ion extraction for chromatogram (± 10 ppm)
	8-nitroG: 195.0266 > 178.0005
	[$^{13}C_2$,^{15}N]-8-nitroG: 198.0304 > 181.0039

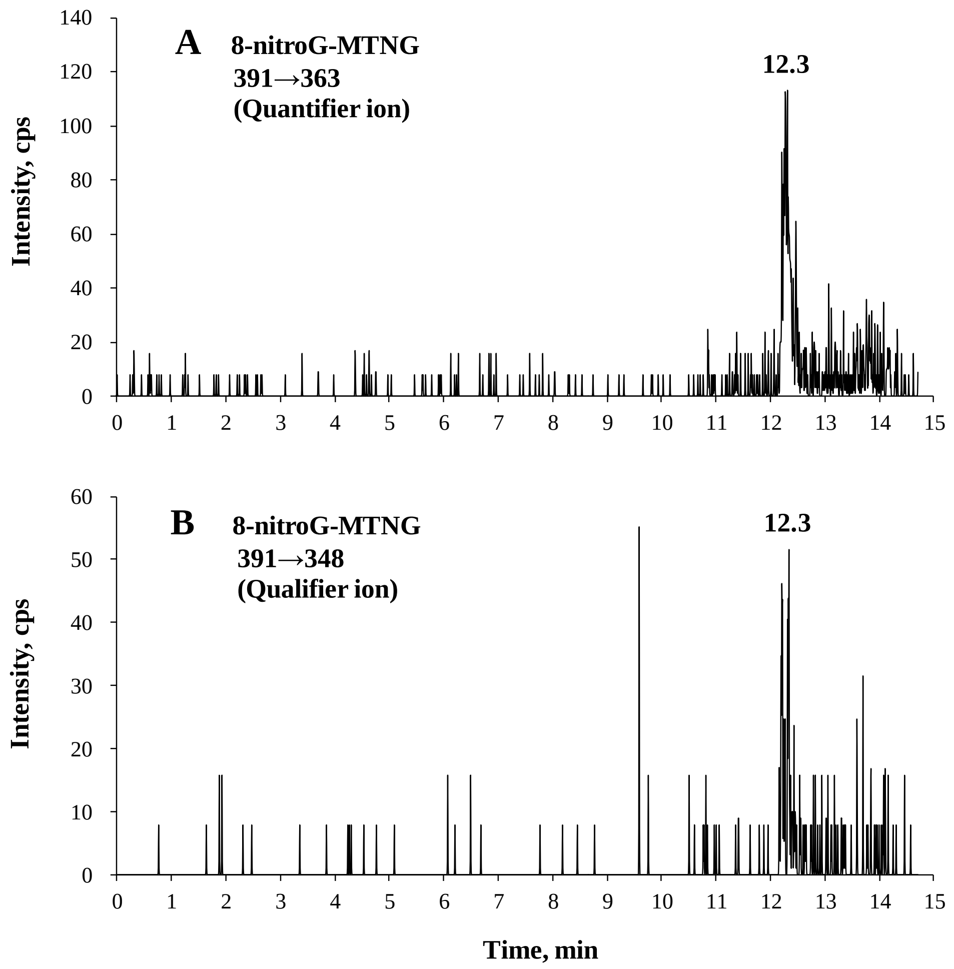

Figure S1. A MRM chromatogram obtained by injection of a 8-nitroG-MTNG sample at 0.015 nM that gave S/N ratios > 3 for both quantifier and qualifier ions.

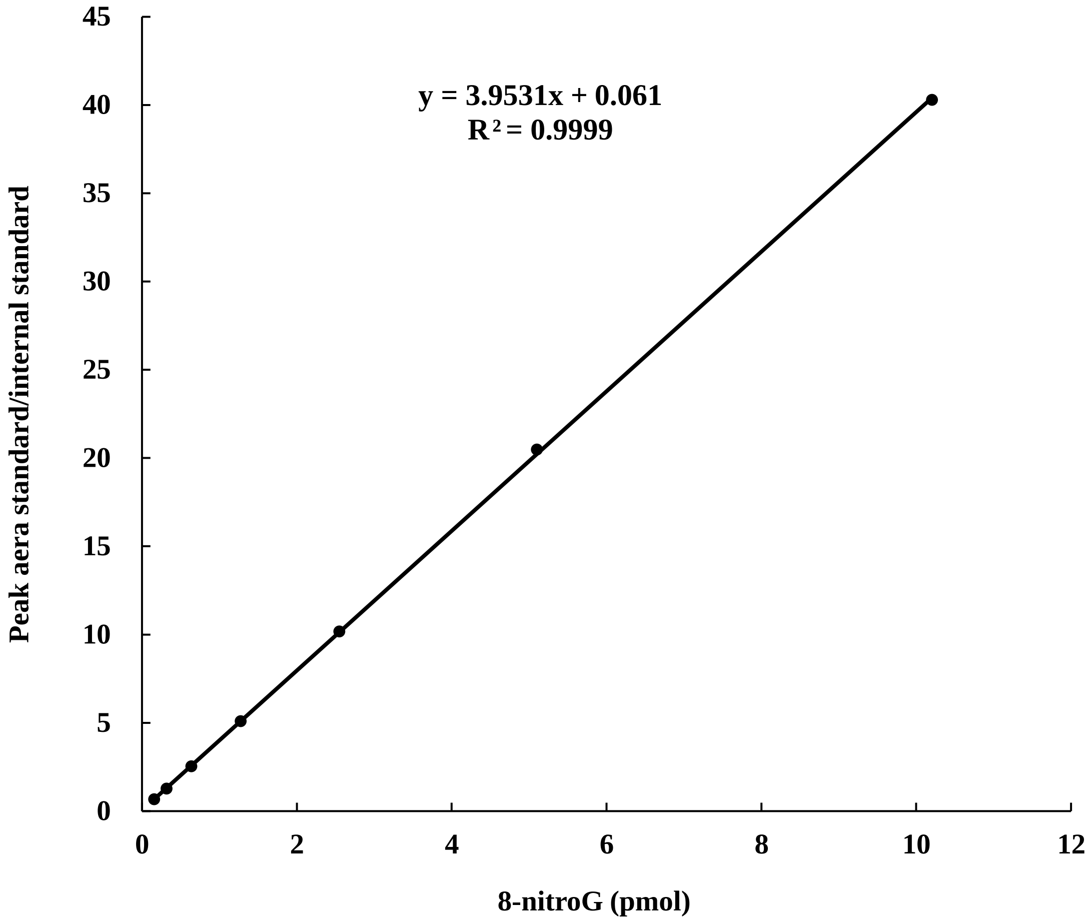

Figure S2. Calibration curve obtained by plotting the peak area ratios of the derivatized analyte (8-nitroG-MTNG) to the derivatized internal standard ([$^{13}C_2$,^{15}N]-8-nitroG-MTNG) as a function of the amount of analyte (8-nitroG). Linear regression calculations were unweighted and non-zero-forced.

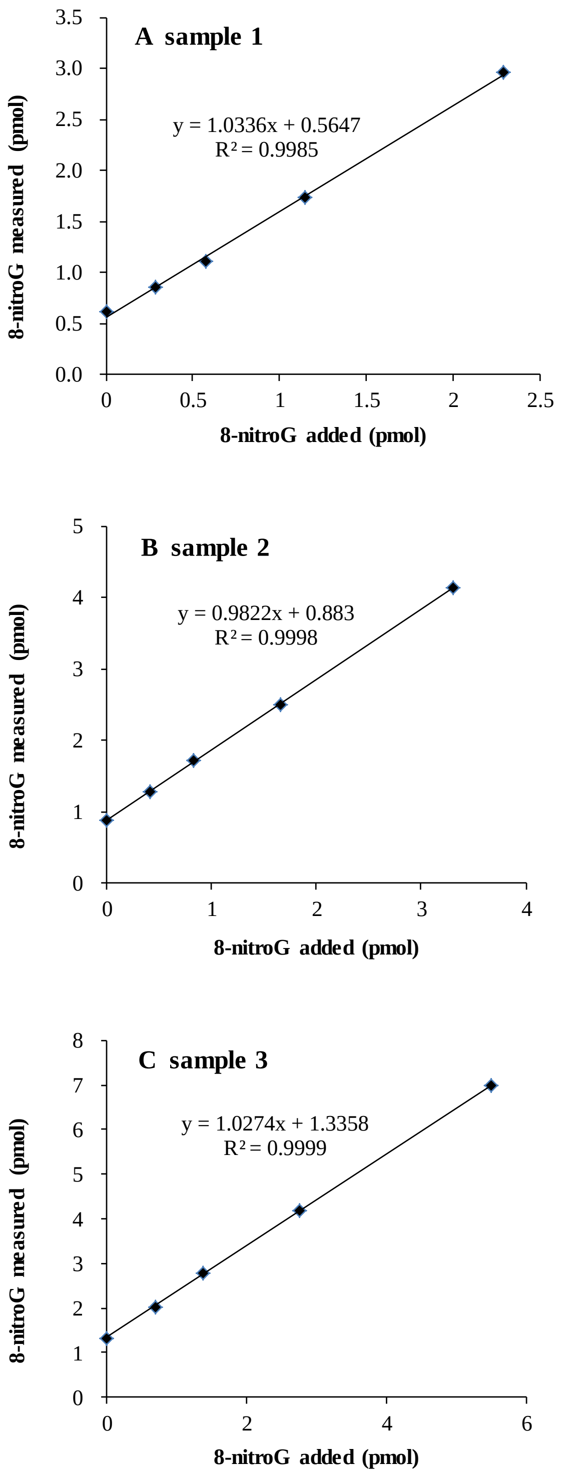

Figure S3. Recovery of isotope-dilution LC-MS/MS with online SPE for 8-nitroG analysis. The calf thymus DNA samples were treated with peroxynitrite at three different concentrations (50 μM for sample 1, 100 μM for sample 2 and 200 μM for sample 3). Recoveries of 8-nitroG were estimated by the addition of unlabeled 8-nitroG standard at 5 different concentrations to sample 1 (A), sample 2 (B) and sample 3 (C).

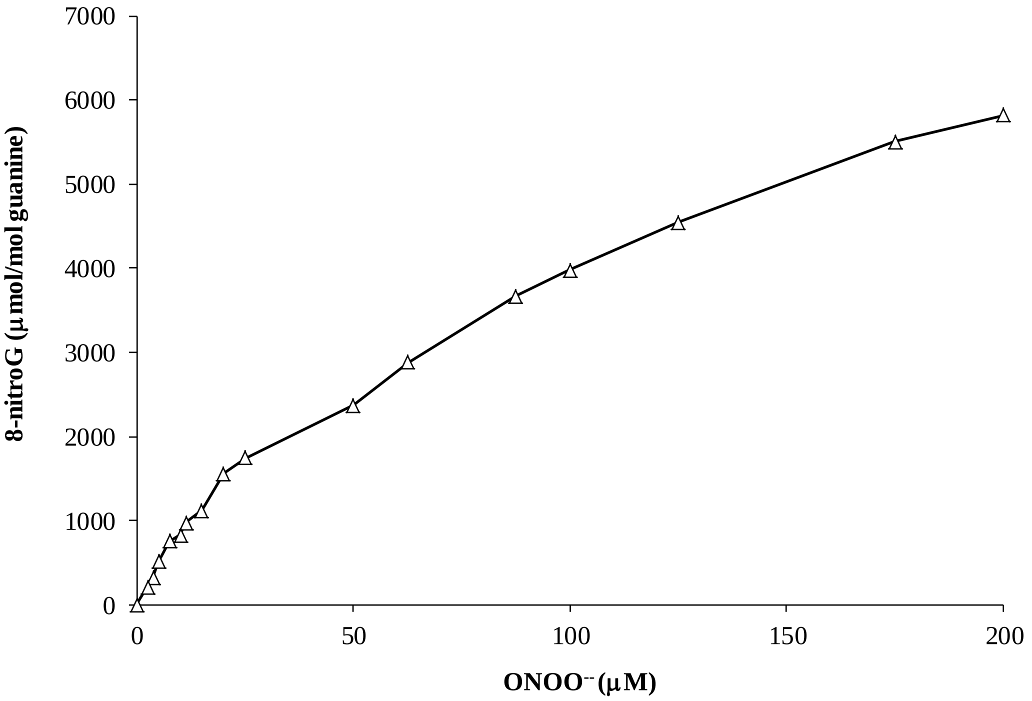

Figure S4. Dose-response analysis of 8-nitroG in peroxynitrite-treated DNA. Calf thymus DNA was incubated with 2.5–200 µM peroxynitrite at pH 7.4. The results are presented as the mean values of duplicate incubations.

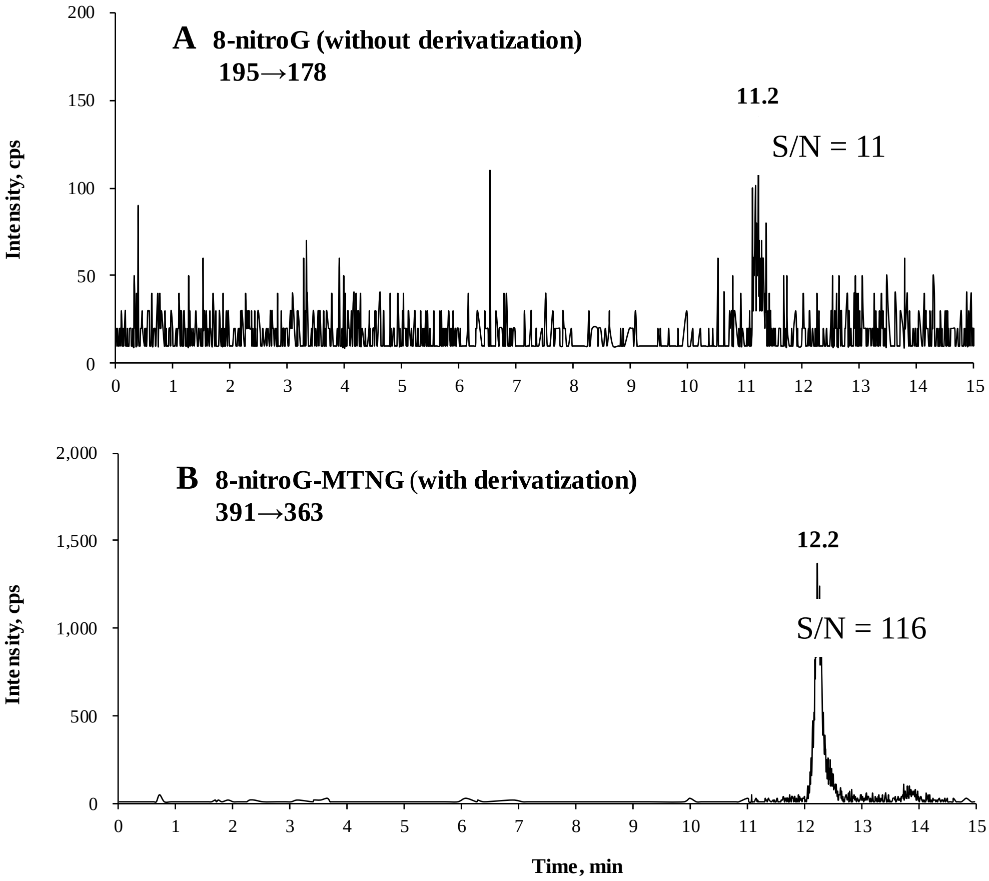

Figure S5. Comparison between the online SPE LC-MS/MS chromatograms without derivatization (A) and with MTNG derivatization (B). Calf thymus DNA was incubated with 5 μM $ONOO^-$, hydrolyzed and divided into two aliquots. One aliquot was directly measured by online SPE LC-MS/MS (22), and the other aliquot was further derivatized with MTNG as described in Materials and Methods prior to online SPE LC-MS/MS analysis. When the sample was measured by the direct LC-MS/MS method, the signal had a signal-to-noise (S/N) ratio of 11 (A). When the sample was measured by LC-MS/MS combined with derivatization, the signal had a S/N ratio of 116 (B). The above finding suggested that a much better sensitivity was achieved by the LC-MS/MS method combined with derivatization.

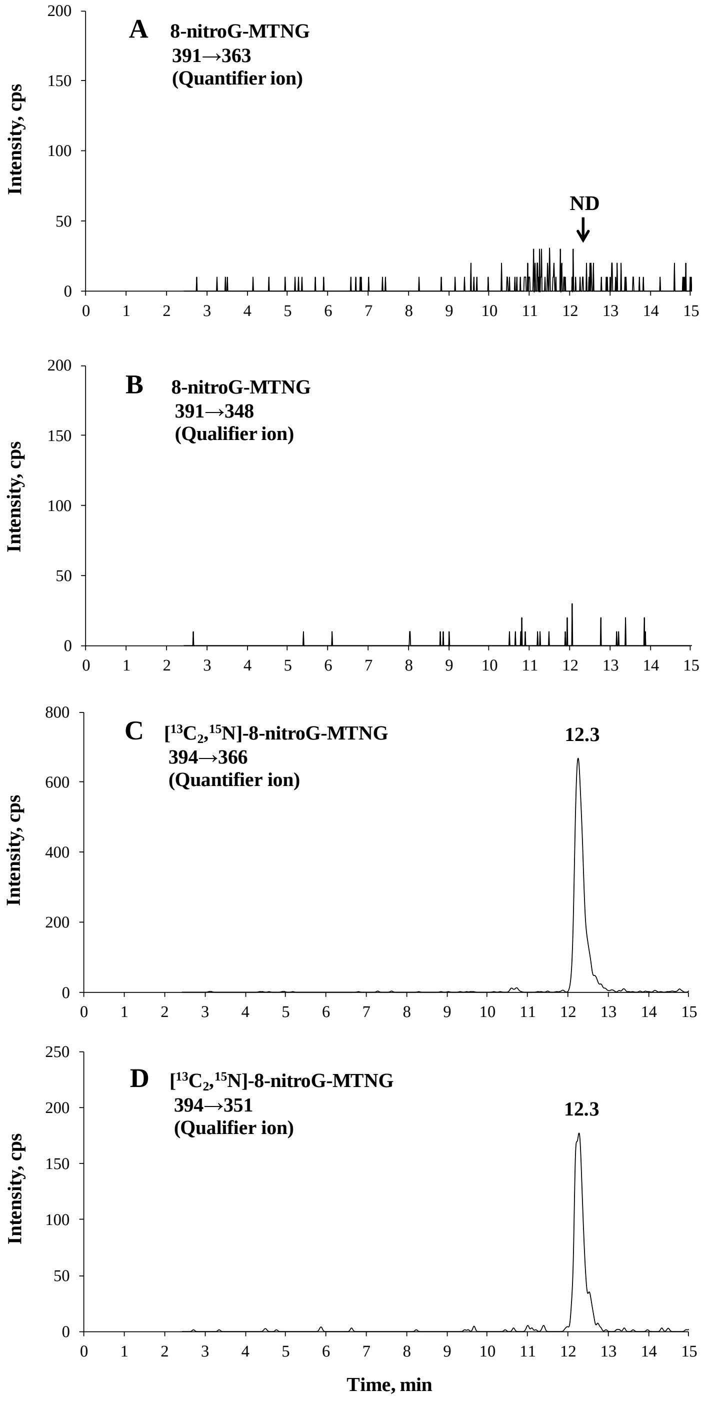

Figure S6. Chromatogram of 8-nitroG-MTNG in the human endothelial hybrid cells (EA. hy-926). The background level of 8-nitroG in cellular DNA was non-detectable (ND).

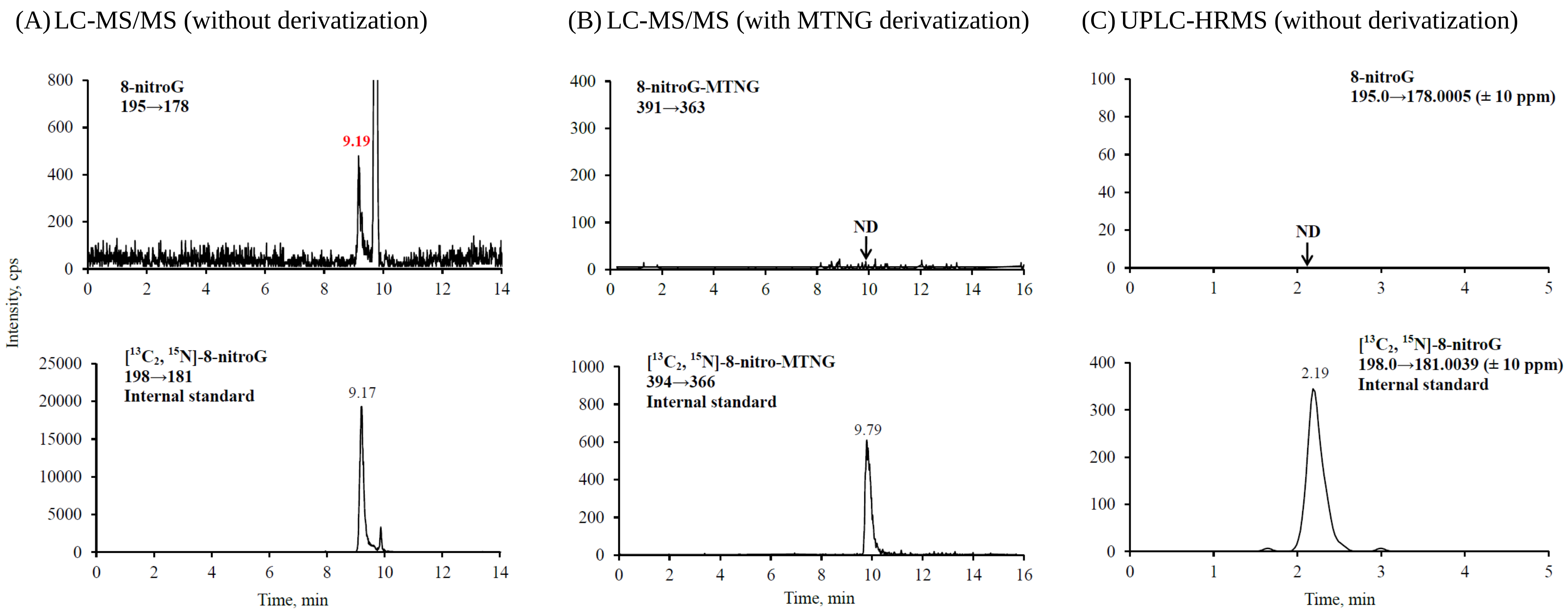

Figure S7. Representative chromatograms obtained from a urine sample as individually measured by LC-MS/MS without derivatization (A), LC-MS/MS with MTNG derivatization (B) and UPLC-HRMS without derivatization (C). One milliliter of urine was pretreated with a manual SPE; the urine was loaded onto a Sep-Pak C18 cartridge (100 mg/1 mL, Waters) preconditioned with methanol and deionized water. The cartridge was directly eluted with 1 mL of 40% methanol containing 1% formic acid. The eluate was dried under vacuum, redissolved in 5% methanol and was further analyzed with or without pre-derivatization. The LOD of LC-MS/MS without derivatization was 0.2 nM in urine (A), while the LOD of LC-MS/MS with MTNG derivatization was 0.01 nM in urine (B). The LOD of the UPLC-HRMS method was 0.1 nM in urine (C). For the same urine sample, a false-positive signal at 9.19 min having the same transition (m/z 195→178) as 8-nitroG was clearly detected

by LC-MS/MS without derivatization (A), whereas no 8-nitroG was detected by the LC-MS/MS with MTNG derivatization (B) or UPLC-HRMS without derivatization (C). This study was approved by the Institutional Review Board of Chung Shan Medical University Hospital. Written informed consent was obtained from each participant before urine sample collection. A total of 10 healthy subjects were recruited into the study, including 6 male smokers and 4 male nonsmokers. A questionnaire was used to obtain data on subject age, body mass index (BMI) and smoking status. The mean age and BMI of the subjects were 55 ± 4.8 years and 24 ± 2.8 kg/m^2, respectively.
